# GM–CSF modulates conserved ILC–like transcriptional states and myeloid cell signaling during ulceration in Crohns disease

**DOI:** 10.64898/2026.02.03.703526

**Published:** 2026-10-14

**Authors:** Joshua K. Morrison, Ksenija Sabic, Neha Maskey, Sayali Talware, Nai-yun Hsu, Colleen Chasteau, Elizabeth Aslinger, Jake T Herb, Shikha Nayar, Rachel Levantovsky, Christopher Tastad, Rachel Moss, Alan Soto, Monica Garcia-barros, Jessy Ntunzwenimana, Mariel Glass, Michelle Bao, Jiayu Zhang, Kyle Gettler, Huajun Han, Jane Stevens, Lorena Tavares, Tin Htwe Thin, Sergey Khaitov, Alexander Greenstein, Rachel Brody, Jaime Chu, Arthur Mortha, Judy H Cho, Ling-shiang Chuang

## Abstract

Macrophage (M-), granulocyte (G-), and granulocyte-macrophage (GM-) colony-stimulating factors (CSFs) regulate myeloid cell function, yet their relative roles during inflammation remain poorly defined. To investigate how CSFs are spatially organized in Crohn's disease, we performed Xenium single-cell spatial transcriptomics on ileal tissues, revealing cell-type-specific expression and source-target interactions for each CSF. GM-CSF, unlike M-CSF or G-CSF, was locally enriched in ulcerated regions and associated with STAT5 phosphorylation within macrophage aggregates. To study functional consequences of GM-CSF signaling, we developed a csf2rb⁻/⁻ zebrafish model of intestinal injury. Loss of GM-CSF signaling exacerbated epithelial damage and inflammation, whereas recombinant human GM-CSF limited injury and was associated with a reduced DSS-induced increase in putative ILC1-like cells, preservation of putative ILC3-like cells, and increased IL-22 production. Cross-species single-cell analysis revealed conserved ILC-like transcriptional states and GM-CSF-responsive myeloid transcriptional programs. Together, these findings demonstrate a protective role for GM-CSF during intestinal inflammation and support a model in which spatially localized GM-CSF signaling and associated myeloid and ILC-like cell states contribute to intestinal immune homeostasis in Crohn's disease.

## Full Text Availability

The license terms selected by the author(s) for this preprint version do not permit archiving in PMC. The full text is available from the preprint server.

